# Multivariate statistical analysis of bioavailability of heavy metals and mineral characterization in selected species of coastal flora

**DOI:** 10.1038/s41598-024-62201-0

**Published:** 2024-05-17

**Authors:** Tarla Chudasama, Kiran Dangar, Kamlesh Gadhvi, Suhas Vyas, Dushyant Dudhagara

**Affiliations:** 1 Department of Life Sciences, School of Science, Dr. Subhash University, Junagadh, Gujarat India; 2 Savaj Junagadh Dist. Co-Operative Milk Producers Union Ltd, Junagadh, Gujarat India; 3 Gujarat Forestry Research Foundation, Gandhinagar, Gujarat India; 4 Department of Life Sciences, Bhakta Kavi Narsinh Mehta University, Junagadh, Gujarat India

**Keywords:** Heavy metals, Mineral composition, Coastal flora, Principal component analysis (PCA), Bioavailability, Environmental monitoring, Ecology, Physiology, Ecology, Environmental sciences

## Abstract

This study presents a thorough investigation into the concentration of heavy metals and mineral composition within four distinct coastal flora species: *Cyperus conglomeratus, Halopyrum mucronatum, Sericostem pauciflorum*, and *Salvadora persica*. Employing rigorous statistical methodologies such as Pearson coefficient correlation, principal component analysis (PCA), analysis of variance (ANOVA), and interclass correlation (ICC), we aimed to elucidate the bioavailability of heavy metals, minerals, and relevant physical characteristics. The analysis focused on essential elements including copper (Cu), iron (Fe), manganese (Mn), zinc (Zn), magnesium (Mg^2+^), calcium (Ca^2+^), sodium (Na^+^), potassium (K^+^), and chloride (Cl^–^), all of which are known to play pivotal roles in the ecological dynamics of coastal ecosystems. Through PCA, we discerned distinctive patterns within PC1 to PC4, collectively explaining an impressive 99.65% of the variance observed in heavy metal composition across the studied flora species. These results underscore the profound influence of environmental factors on the mineral composition of coastal flora, offering critical insights into the ecological processes shaping these vital ecosystems. Furthermore, significant correlations among mineral contents in *H. mucronatum*; K^+^ with content of Na^+^ (r = 0.989) and Mg^2+^ (r = 0.984); as revealed by ICC analyses, contributed to a nuanced understanding of variations in electrical conductivity (EC), pH levels, and ash content among the diverse coastal flora species. By shedding light on heavy metal and mineral dynamics in coastal flora, this study not only advances our scientific understanding but also provides a foundation for the development of targeted environmental monitoring and management strategies aimed at promoting the ecological sustainability and resilience of coastal ecosystems in the face of ongoing environmental challenges.

## Introduction

The word halophyte has been coined and introduced in the language of botany since the mid-nineteenth century, as it has been well established in the last decade^1^. Halophytes are salt-resistant or salt-tolerant plants and have a remarkable ability to complete their life cycle in saline conditions. During evolution, they have developed different morphological, anatomical and physiological strategies to proliferate in high-salt environments^2–12^. Some investigations reflect that some salt-tolerant species require salt content for their growth^13–15^. Na^+^/H^+^ exchange was significantly higher in vesicles made from leaves and increased with plant salinity^16^. Halophytes require appropriate standardization of Na^+^ and K^+^ concentrations inside their cells, referring to a resistance mechanism to hydroxyl radicals^17^. The response of plants to natural environmental behavior differs from that of saline strains with combined stress alone^6^. The regulation of salt tolerance capacity is mainly based on the connection or association between salt and water. Halophytes are able to reduce the accumulation of salts in the solution in the plant tissue due to which the amount of salt liberated to the leaves is absorbed by the salivary glands through growth and ion secretion^18^. Furthermore, the study of halophytic species has broader implications beyond their ecological adaptations. Understanding their mechanisms of salt tolerance and the intricate balance of ions within their cells can inspire innovative approaches in agriculture, bioengineering, and bioremediation. Halophytes offer potential solutions for addressing soil salinity issues in agriculture, as well as for reclaiming and rehabilitating degraded saline soils. Moreover, their unique physiological traits may hold promise for developing drought-tolerant crops and improving saline agriculture practices in arid and semi-arid regions. Halophytes, especially from many plant species, are capable of accumulating large amounts of salts in sprouting tissues. Many researchers have carried out the study of some halophytic species to investigate the salt tolerance or requirement for growth and development and it concluded that in natural saline habitats, salt essentially does not affect the growth of salt-tolerant species but also was a great factor of ionic constitutes and other components of the coastal flora^13^. In addition, the ecological importance of halophytes extends to their role in coastal protection and ecosystem services. Their ability to stabilize coastal soils, mitigate erosion, and provide habitat and food sources for wildlife contributes to the resilience of coastal ecosystems in the face of climate change and sea-level rise. By studying halophytes and their interactions with their saline environments, researchers can gain insights into the functioning and dynamics of coastal ecosystems, guiding conservation efforts and sustainable management practices.

Various Halophytes are high salt tolerance capacities such as *Halopyrum mucronatum, Cyperus conglomeratus, Polycarpaea spicata*^19^, *Cressa cretica, Haloxylon stocksii, Suaeda fruticosa* and *Halopyrum mucronatum*^20^, and *Halopyrum mucronatum* and *Cenchrus ciliaris*^21^ halophytic plant expressed differential response to water deficit. Some plants and halophytes have adaptation or resistance to saline or brackish irrigation conditions with a salinity ratio of seawater. Approximately 72% of the earth’s surface is covered by saltwater, dominated by Na^+^ and Cl^-^. Usually, excessive concentrations of Na^+^ and Cl^-^ and a substantial concentration of Ca^+2^, K^+^, Mg^2+^, and SO_2_ are found in saline soils. The plant’s growth requires essential nutrients supply are very low but exceptional plants such as halophytes are still surviving in this type of diverse condition, so the halophytes are considered as a flora of saline environment^8,22^. Although some halophytic species are relatively new and still evolving, they have some common characteristics^17^. Multiple stresses are tolerated by halophytic species but the salt-tolerance compartmentalization model suggested the occurrence of the osmotic adjustment with the ions of the environment^23^. Halophytic ion concentrations (Na^+^ and Cl^-^) are exposed to toxicity when a supra-optimal saline condition is not clear. The main factors of the salt tolerance ability of halophytes are mainly based on the regulated uptake control of Na^+^ and Cl^-^ and also maintaining a concentration of cytoplasmic K^+^ and Mg^+^ required for essential enzyme activation^24^. In agricultural contexts, where soil salinity poses a significant challenge to crop productivity, halophytes offer potential solutions for reclaiming and rehabilitating degraded saline soils. Their ability to thrive in saline environments suggests they may possess genes or mechanisms that could be harnessed to improve crop salt tolerance through genetic engineering or selective breeding. Furthermore, halophytes play crucial roles in ecosystem resilience and conservation, particularly in coastal regions. By stabilizing coastal soils, mitigating erosion, and providing habitat and food sources for wildlife, halophytes contribute to the overall health and sustainability of coastal ecosystems. Understanding their ecological functions and interactions can inform conservation strategies and ecosystem management practices aimed at preserving coastal biodiversity and resilience in the face of climate change and sea-level rise. From a broader perspective, the study of halophytes offers insights into the fundamental mechanisms of plant salt tolerance and adaptation. By elucidating how halophytes regulate ion uptake, compartmentalize toxic ions, and maintain essential cellular functions in saline environments, researchers can gain valuable knowledge applicable to various fields, including agriculture, biotechnology, and environmental science.

In an ecosystem, plants are an important component and they transfer abiotic elements to biotic things. Some elements like Cu, Fe, Mn, Zn, etc. are toxic elements; they are harmful to living things as well as environmental prospects. Due to the toxicity, heavy-metal pollution of the environment is a global problem, as escalated concentrations in ecosystems have become a significant ecological hazard due to their harmful effects on living things^25–27^. Urbanization and industrialization of coastal regions have brought metal contamination worldwide; however, Phytoremediation is extensively practiced to treat water and soil pollution. The promising method used for the remediation of heavy metals pollution is considered by halophytes^28^. Some halophytes are treated as significant prospects for phytoremediation because they have a well-built capacity to accumulate metals. Assessment of trace-metal bioaccumulation should be the principal characterization on which to base the evaluation of the potential of plants to activate and accumulate metals from soil and water^29,30^. Halophytes have evolved various strategies for the uptake and accumulation of heavy metals from their surroundings^31^. These strategies include enhanced root metal uptake, metal ion sequestration in vacuoles, and metal complexation with phytochelatins, metallothioneins, and organic acids^32^. The choice of halophyte species depends on the specific heavy metal contamination and environmental conditions. Researchers and environmentalists often select halophytes based on their metal-accumulating capacities and adaptability to the polluted site. Further research and field applications will continue to expand our understanding of their potential in heavy metal remediation.

The study is likely to address several key problems related to the bioavailability of heavy metal and mineral composition in the coastal belt of a selected region. However, investigating the extent of heavy metal contamination in coastal flora and its potential impact on ecosystem health. The bioavailability of heavy metals in the environment determines their uptake and accumulation in plants understanding the factors influencing heavy metal bioavailability in coastal flora is crucial for assessing the risks posed to both plant and human health. The mineral composition of plants plays a vital role in their growth, development, and overall health; by characterizing the mineral content of selected species of coastal flora. The study aims to identify key minerals and their potential role in mitigating heavy metal toxicity or enhancing plant tolerance to metal stress. The study explored the complex interrelationships between heavy metal concentrations and mineral content within coastal flora species. Multivariate statistical analysis techniques such as principal component analysis (PCA) and Pearson coefficient correlation can help identify patterns and associations between heavy metals and minerals, providing insights into the mechanisms governing metal uptake and accumulation in plants. Ultimately, the study aims to contribute valuable information for environmental management and conservation efforts in coastal ecosystems. By identifying hotspots of heavy metal contamination and understanding the factors influencing metal bioavailability and plant-mineral interactions, the study can guide the development of effective pollution control measures and conservation strategies aimed at preserving coastal flora and ecosystem integrity. Overall, the investigation was addressing significant environmental challenges related to heavy metal pollution and mineral composition in coastal flora, with the ultimate goal of informing sustainable management practices and safeguarding coastal ecosystem health.

## Material and methods

### Location of plant sampling

The research was carried out within the coastal region of Junagadh district (1° 13′ N to 21° 00′ N and 69° 59 ′ E to 70° 13′ E), Gujarat, India. The Junagadh district contains an approximate 45 km coastal belt with fishing ports, industries, trade and logistics, waterway interactions as well and forest patches. The coastal belt of Junagadh district was differentiated in three different habitats, sandy, marshy and rocky; majority of coastal belt covered with sandy habitat. More than half of the coastal belt of Junagadh district is covered by forest patches; majorly it consists of *Casuarina equisitifolia* and *Prosopis juliflora*. Up to a distance of 100 m from the sea, the dominant species *Cyperus conglomerates, Halopyrum mucronatum, Sericostem pauciflorum, and Salvadora persica* were observed and selected for the analysis. Plant samples were collected from the selected 12 locations of coastal belt associated with Junagadh district, Gujarat in deferent three seasons winter, monsoon and summer; respectively.

### Permission statement

Some selected sites (for the collection of samples from the coastal belt of Junagadh district) of the research area are covered under protected areas. Therefore, permission was granted by the Department of Forest, Development & Management, Gujarat state.

### Plant guideline statement

All the plant experiments/protocols were performed with relevant institutional, national, and international guidelines and legislation. Experimental research and field studies on plants including a collection of plant materials according to the Indian Biodiversity Act (2002) and methods used for sample collection as per the American Public Health Association, & American Water Works Association^33^.

### Plant specimen identification

Plant species were identified by Dr. Tarla Chudasama and Dr. Kamlesh Gadhvi with the help of Flora of Gujarat state^34^. The voucher specimens (BKNMU 043, 044, 045 and 046) are deposited at the Department of Life Sciences, Bhakta Kavi Narsinh Mehta University, Junagadh, Gujarat.

### Experimental work

Leaves of two monocotyledonous plant species *Cyprus conglomeratus and Halopyrum mucronatum* and two dicotyledonous plant species *Sericostoma pauciflorum and Salvadora persica* were collected during monsoon, winter and summer seasons. The material was washed and dried in room temperature or in oven (60 to 80 °C). 1 g of dried plant material was taken in silica crucible and ashed in burner to complete oxidation of the organic matter and add 1 ml of concentrated nitric acid. The acid was evaporated in water bath and crucible were again placed in burner for complete ashing. After cooling the crucible add 20 ml 1:1 hydrochloric acid and the extract was evaporated in water bath. Final addition of 20 ml distilled water and then extract filtered through Whatman filter paper no. 44 with repeated washing and final volume made up 250 ml with distilled volume. This sample were used for the estimation of calcium (Ca^2+^) and magnesium (Mg^2+^) by EDTA (ethylene diamine tetra acetic acid) titration, sodium (Na^+^) and potassium (K^+^) by flame photometry (Systronics-130 India) method^35^.

1 g of dried plant material was boiled in 100 ml of deionized water in a water bath for 1 h and the extract was filtered. A filtered sample was used for the estimation of chloride (Cl^-^) by the argentometric method^36^, pH through pH meter (Eutech India) and Electrical conductivity for EC meter (Systronics-306 India).

### Multivariate statistical analysis

The analysis of bioavailability of heavy metal and mineral characterization in coastal flora, Pearson coefficient correlation, principal component analysis (PCA), analysis of variance (ANOVA) and interclass correlation (ICC) were performed using commercial software (SPSS). The coefficient correlation measured the interactive strength of two variables. PCA and ICC are the incredibly usual multivariate statistical methods used in environmental research^37^.

## Results

### Principle component analysis (PCA)

The Principal Component Analysis (PCA) on the loading of heavy metals on *Halopyrum mucronatum*, highlighting the significant findings (Table 1). The PCA aimed to identify the main variables contributing to the variance in heavy metal concentrations. Four principal components (PCs) were extracted and analyzed for their respective loadings of each metal. The first principal component (PC1) emerges as the most significant, with an eigenvalue of 4.88899. This component explains a substantial proportion of the total variance in the dataset. Notably, Cu (− 0.00993) and Fe (0.99995) exhibit the highest loadings on PC1, indicating a strong correlation between these metals. Their association suggests that variations in Cu and Fe concentrations in *Halopyrum mucronatum* are closely linked. PC2, while contributing a relatively smaller amount to the overall variance, still provides valuable insights. Mn (0.61695) and Zn (0.76142) display the highest loadings on PC2, indicating a positive correlation between these metals. The relationship between Mn and Zn concentrations becomes noteworthy, although their influence on the overall variance is less pronounced compared to PC1. Regarding PC3, with an eigenvalue of 0.00233158, it contributes to the variance to a lesser extent. Nonetheless, Mn (0.78584) shows the highest loading on PC3, emphasizing its significance in explaining the variance along this component. Finally, PC4 demonstrates the smallest eigenvalue of 0.00017976, suggesting the least contribution to the overall variance. However, Cu (0.97449) exhibits the highest loading on PC4, indicating a strong influence of Cu on this component. Overall, the PCA results highlight the significant role of Cu, Fe, Mn, and Zn as the primary heavy metals contributing to the observed variance in *Halopyrum mucronatum*. Cu and Fe exhibit the most substantial impact, as evidenced by their contributions to the highest eigenvalue and loadings on PC1.

**Table 1:** PCA loading of heavy metals on *Halopyrum mucronatum*.

Variables	PC 1	PC 2	PC 3	PC 4
Cu	− 0.0099294	0.19901	− 0.1033	**0.97449**
Fe	**0.99995**	0.0019527	0.0020116	0.010003
Mn	− 0.0023583	0.61695	0.78584	− 0.042716
Zn	0.0019417	0.76142	− 0.60974	− 0.22012
Eigenvalue	4.88899	0.0144954	0.00233158	0.00017976
% variance	99.653	0.29546	0.047525	0.0036641

Significant values are in bold.

The significance of the study lies in its ability to elucidate the intricate relationships between heavy metal concentrations in *Halopyrum mucronatum*, a plant species often used in phytoremediation efforts due to its metal tolerance. By employing Principal Component Analysis (PCA), the study identifies the key variables contributing to the variance in heavy metal concentrations within the plant. The findings underscore the prominent roles of copper (Cu), iron (Fe), manganese (Mn), and zinc (Zn) in influencing the observed variance. Specifically, the first principal component (PC1) emerges as the most significant, with Cu and Fe exhibiting the highest loadings. This indicates a strong correlation between Cu and Fe concentrations in *Halopyrum mucronatum*, suggesting that variations in these metals are closely linked within the plant. Moreover, while PC2, PC3, and PC4 contribute to the variance to a lesser extent, they still provide valuable insights into the relationships between other heavy metals such as Mn and Zn. These findings offer a comprehensive understanding of the interplay between different heavy metals within the plant species. The significance of this study extends beyond the identification of heavy metal concentrations; it provides crucial insights for environmental scientists, agronomists, and policymakers involved in phytoremediation and environmental management. Understanding the dynamics of heavy metal accumulation and correlation within specific plant species like *Halopyrum mucronatum* is vital for designing effective remediation strategies and mitigating the impacts of metal pollution on ecosystems and human health. Furthermore, the study's methodology showcases the applicability of PCA in unraveling complex datasets, thereby contributing to the broader field of environmental science and analytical chemistry.

In *Cyprus conglomeratus,* the principal component analysis (PCA) conducted on the loading of heavy metals and highlighting the significant findings (Table 2). The objective of the PCA was to identify the main variables contributing to the variance in heavy metal concentrations. The table presents the loadings for each metal across four principal components (PCs). The first principal component (PC1) emerges as the most significant, as it possesses the highest eigenvalue of 1.03602. PC1 explains a substantial proportion of the total variance in the dataset. Cu (0.0099916) exhibits the highest loading on PC1, indicating a positive correlation between Cu concentrations and the variance observed in *Cyprus conglomeratus*. This suggests that changes in Cu levels are likely to have a significant impact on the overall variability of heavy metal concentrations in this plant species. PC2, with an eigenvalue of 0.0181766, contributes to a notable portion of the overall variance. Fe (0.99484) demonstrates the highest loading on PC2, implying a strong positive correlation between Fe concentrations and the variance observed along this component. This finding suggests that variations in Fe levels may contribute significantly to the observed differences in heavy metal concentrations in *Cyprus conglomeratus*. PC3, with an eigenvalue of 0.00278129, contributes to the variance to a lesser extent. Mn (0.52262) displays the highest loading on PC3, indicating its significance in explaining the variance along this component. The positive loading suggests that changes in Mn concentrations are associated with the observed variability in heavy metal levels in *Cyprus conglomeratus*. Similarly, PC4 exhibits a relatively smaller eigenvalue of 0.000234194, indicating its minimal contribution to the overall variance. Zn (0.83823) demonstrates the highest loading on PC4, suggesting a positive correlation between Zn concentrations and the observed variance. The loading indicates that changes in Zn levels may have a lesser but still notable impact on the overall variability of heavy metal concentrations in *Cyprus conglomeratus*. Overall, the PCA results highlight the significant role of Cu, Fe, Mn, and Zn as the primary heavy metals contributing to the observed variance in *Cyprus conglomeratus*. Cu and Fe exhibit the most substantial impact, as evidenced by their contributions to the highest eigenvalue and loadings on PC 1 and PC 2, respectively.

**Table 2:** PCA loading of heavy metals on *Cyprus conglomeratus*.

Variables	PC 1	PC 2	PC 3	PC 4
Cu	0.0099916	0.029147	0.12123	0.99215
Fe	0.99484	− 0.027193	− 0.097703	0.0027187
Mn	0.074656	0.84457	0.52262	− 0.089423
Zn	0.067966	− 0.53396	0.83823	− 0.087423
Eigenvalue	1.03602	0.0181766	0.00278129	0.000234194
% variance	97.995	1.7193	0.26308	0.022152

The significance of the study on heavy metal concentrations in *Cyprus conglomeratus*, as determined through Principal Component Analysis (PCA), is multifaceted and holds implications for both environmental science and ecological management. Firstly, by identifying the main variables contributing to the variance in heavy metal concentrations within Cyprus conglomeratus, this study offers critical insights into the dynamics of heavy metal accumulation in this plant species. Understanding which metals have the most substantial impact on the observed variability allows for targeted monitoring and remediation efforts in environments where *Cyprus conglomeratus* grows. The findings highlight the significant roles of copper (Cu), iron (Fe), manganese (Mn), and zinc (Zn) in influencing the observed variance. Notably, the strong positive correlation between Cu concentrations and the variance observed in PC1 suggests that changes in Cu levels may serve as indicators for overall heavy metal variability in *Cyprus conglomeratus*. Similarly, the high loading of Fe on PC2 underscores the importance of iron concentrations in explaining differences in heavy metal levels within the plant species. This analytical approach can be valuable for researchers and environmental practitioners involved in assessing metal pollution and devising effective mitigation strategies.In a broader context, understanding heavy metal accumulation in *Cyprus conglomeratus* contributes to our knowledge of plant-metal interactions and phytoremediation potential. This knowledge can inform decisions regarding the use of *Cyprus conglomeratus* in environmental restoration projects aimed at mitigating metal contamination in soils and water bodies. Overall, the study provides valuable insights into the factors influencing heavy metal concentrations in *Cyprus conglomeratus*, thereby contributing to the advancement of environmental science and the development of sustainable strategies for managing metal pollution in natural ecosystems.

The PCA conducted on the loading of heavy metals on *Sericostoma pauciflorum*, shedding light on the significant findings (Table 3). The PCA aimed to identify the main variables contributing to the variance in heavy metal concentrations. The table displays the loadings for each metal across four principal components (PCs). The first principal component (PC1) stands out as the most significant, featuring the highest eigenvalue of 1.31452. PC1 explains a substantial proportion of the total variance in the dataset. Cu (0.008826) exhibits the highest loading on PC1, indicating a positive correlation between Cu concentrations and the observed variance in *Sericostoma pauciflorum*. This suggests that variations in Cu levels are likely to have a significant impact on the overall variability of heavy metal concentrations in this species. PC2, with an eigenvalue of 0.0118029, contributes to a relatively smaller portion of the overall variance. Mn (0.98898) demonstrates the highest loading on PC2, implying a strong positive correlation between Mn concentrations and the variance observed along this component. This finding suggests that variations in Mn levels may contribute significantly to the observed differences in heavy metal concentrations in *Sericostoma pauciflorum*. PC3, with an eigenvalue of 0.000276787, contributes to the variance to a lesser extent. Cu (0.78811) displays the highest loading on PC3, indicating its significance in explaining the variance along this component. The positive loading suggests that changes in Cu concentrations are associated with the observed variability in heavy metal levels in *Sericostoma pauciflorum*. Similarly, PC4 exhibits a relatively smaller eigenvalue of 7.49223E-05, indicating its minimal contribution to the overall variance. Zn (0.79132) demonstrates the highest loading on PC4, suggesting a positive correlation between Zn concentrations and the observed variance. The loading indicates that changes in Zn levels may have a lesser but still notable impact on the overall variability of heavy metal concentrations in *Sericostoma pauciflorum*.

**Table 3:** PCA loading of heavy metals on *Sericostoma pauciflorum*.

Variables	PC 1	PC 2	PC 3	PC 4
Cu	0.008826	0.081768	0.78811	0.61001
Fe	0.99857	0.047567	− 0.022904	0.0087672
Mn	− 0.050519	0.98898	− 0.13324	0.0403
Zn	− 0.015294	− 0.11393	− 0.6005	0.79132
Eigenvalue	1.31452	0.0118029	0.000276787	7.49223E− 05
% variance	99.084	0.88966	0.020863	0.0056474

The principal component analysis (PCA) conducted on the loading of heavy metals in *Sericostoma pauciflorum* provides critical scientific insights into the intricate dynamics of metal accumulation within this aquatic species. By identifying copper (Cu) and manganese (Mn) as the primary variables contributing to the observed variance, the study elucidates the major drivers of heavy metal concentrations in *Sericostoma pauciflorum* populations. The strong positive correlation between Cu concentrations and the variance observed in PC1 underscores the significant role of copper in influencing overall heavy metal variability, while the substantial loading of Mn on PC2 highlights the importance of manganese concentrations in explaining differences in metal levels within the species. These findings deepen our understanding of metal dynamics in marine water habitats where *Sericostoma pauciflorum* resides, offering valuable insights into environmental quality and potential contamination sources. Moreover, the application of PCA in this study showcases its utility as an analytical tool for unraveling complex datasets in environmental science, providing a methodological framework for future research endeavors aimed at understanding and mitigating metal pollution in aquatic ecosystems. Ultimately, these findings contribute to the broader scientific knowledge base concerning environmental pollution and offer insights that can inform management and conservation efforts aimed at preserving the health and biodiversity of marine water ecosystems.

In *Salvadora persica*, the PCA conducted on the loading of heavy metals and providing valuable insights into the significant findings (Table 4). The PCA aimed to identify the main variables contributing to the variance in heavy metal concentrations. The table displays the loadings for each metal across four principal components (PCs). The first principal component (PC1) emerges as the most significant, explaining a substantial proportion of the total variance with a percentage of the variance of 77.592%. Cu (− 0.1615) exhibits the highest loading on PC 1, indicating a negative correlation between Cu concentrations and the observed variance in *Salvadora persica*. On the other hand, Fe (0.98456) shows a strong positive loading on PC1, suggesting a positive correlation between Fe concentrations and the variance. These findings suggest that Cu and Fe concentrations contribute significantly to the observed variability in heavy metal levels in *Salvadora persica*. PC2, with a percentage of variance of 21.985%, contributes a notable portion to the overall variance. Cu (0.98487) exhibits the highest loading on PC2, indicating its significance in explaining the variance along this component. The positive loading suggests a positive correlation between Cu concentrations and the variance observed along PC2. In PC3, with a percentage of variance of 0.42223%, contributes to the variance to a lesser extent. Mn (0.59021) displays the highest loading on PC 3, indicating its importance in explaining the variance along this component. The positive loading suggests a positive correlation between Mn concentrations and the observed variability in heavy metal levels in *Salvadora persica*.

**Table 4:** PCA loading of heavy metals on *Salvadora persica*.

Variables	PC 1	PC 2	PC 3
Cu	− 0.1615	0.98487	− 0.0404
Fe	0.98456	0.15971	− 0.06825
Mn	0.059552	− 0.005435	0.59021
Zn	0.031772	0.067091	0.80334
Eigenvalue	0.216601	0.0613727	0.00117867
% variance	77.592	21.985	0.42223

The Principal Component Analysis (PCA) study on heavy metal loadings in *Salvadora persica* holds significant implications for marine environment soil dynamics. While Salvadora persica primarily thrives in terrestrial habitats, its proximity to coastal areas and ability to tolerate saline soils make it a valuable indicator species for understanding metal accumulation in environments adjacent to marine ecosystems. By identifying copper (Cu), iron (Fe), manganese (Mn), and other heavy metals as key variables contributing to the observed variance, the study offers insights into the potential pathways through which metals can enter marine ecosystems. Moreover, understanding how Salvadora persica accumulates heavy metals provides valuable information for assessing the risk of metal transfer to marine organisms through ecological pathways, including bioaccumulation and biomagnification. The findings also highlight the potential for *Salvadora persica* to serve as a tool for phytoremediation efforts in coastal soils, thus reducing the risk of metal leaching into marine environments. By incorporating data from terrestrial plants like *Salvadora persica* into integrated coastal zone management plans, policymakers can develop more effective strategies for preserving coastal biodiversity and mitigating the impacts of pollution on marine environments. Ultimately, this study contributes to our broader understanding of the interconnectedness between terrestrial and marine ecosystems and informs efforts to safeguard the health and integrity of coastal environments.

### Pearson coefficient correlation

The results (Table 5) of the Pearson correlation in the ionic composition of *H. mucronatum* leaf indicated that the negative and non-significant (P > 0.05) correlation observed in Cl^–^ with Ca^2+^ (r = − 0.645), Mg^2+^ (r = − 0.684), Na^+^ (r = − 0.751) and K^+^ (− 0.720). The concentration of Ca^2+^ was positive and significantly (P < 0.05) correlated with Na^+^ (r = 0.800), K^+^ (r = 0.856) and Mg^2+^ (r = 0.840); however, the concentration of Mg^2+^ *H. mucronatum* leaf was positive and highly significant (P < 0.01) with Na^+^ (r = 0.971) and K^+^ (r = 0.984). Although, a positive and highly significant correlation was observed between the amount of Na^+^ with the K^+^ (r = 0.989) in this plant leaf sample. The described results denoted that the *H. mucronatum* leaf ionic composition was internally positive and significantly correlated with each other in except for the Cl^–^ content. The concentration of Cl^–^ in this plant leaf was not significantly correlated with the Ca^2+^, Mg^2+^, Na^+^ and K^+^; it was noted that the concentration of the Cl^–^ was negative and non-significant with other elements.

**Table 5:** Pearson correlation in ionic composition of coastal flora (*H. mucronatum, C. conglomeratus, S. pausiflorum* and *S. persica*) leaf.

Plant species		Cl^−^	Ca^2+^	Mg^2+^	Na^+^	K^+^
*H. mucronatum*	Cl^−^	1				
	Ca^2+^	− 0.645	1			
	Mg^2+^	− 0.684	0.840*	1		
	Na^+^	− 0.751	0.800*	0.971**	1	
	K^+^	− 0.720	0.856*	0.984**	0.989**	1
*C. conglomeratus*	Cl^−^	1				
	Ca^2+^	− 0.348	1			
	Mg^2+^	0.016	− 0.044	1		
	Na^+^	0.465	− 0.483	0.072	1	
	K^+^	0.123	− 0.226	0.413	0.708*	1
*S. pausiflorum*	Cl^–^	1				
	Ca^2+^	− 0.078	1			
	Mg^2+^	0.391	− 0.012	1		
	Na^+^	0.247	− 0.499	− 0.325	1	
	K^+^	0.658	0.579	0.225	0.065	1
*S. persica*	Cl^−^	1				
	Ca^2+^	0.054	1			
	Mg^2+^	0.648	0.494	1		
	Na^+^	0.038	− 0.615	0.218	1	
	K^+^	0.608	0.265	0.039	− 0.716*	1

* Significant at ≤ 0.05.

** Highly significant at ≤ 0.01.

In the *C. conglomeratus,* the positive and significant (P < 0.01) correlation observed between Na^+^ with K^+^ (r = 0.708); However, the positive but non-significant (P > 0.05) correlation was observed with Cl^–^ (r = 0.465) and Mg^2+^ (r = 0.072) and negative non-significant correlated with Ca^2+^ (r = − 0.483). The Cl^–^ content of *C. conglomeratus* leaves was negatively non-significant with Ca^2+^ (r = − 0.348) when the positively non-significant with Na^+^ (r = 0.465), K^+^ (r = 0.123) and Mg^2+^ (r = 0.016). The Pearson correlation analysis of *C. conglomeratus* leaf parameters showed that the leaf Ca^2+^ showed negative and non-significant (P > 0.05) relationship with other ions viz., Na^+^ (r = − 0.483), K^+^ (r = − 0.226), Mg^2+^ (r = − 0.044) and Ca^2+^ as described earlier. Also, a positive and non-significant (P > 0.05) correlation was observed in the concentration of Mg^2+^ with Na^+^ (r = 0.072) and K^+^ (r = 0.413) of this plant leaf. These results mentioned the mean values of *C. conglomeratus* leaf samples during the winter, monsoon and summer seasons. Based on the observations of the Pearson correlation of this plant leaf, it described that only Na^+^ significantly correlated with K^+^; rest of the elements not significant correlation observed in this plant species.

The Pearson correlation of ionic composition of the *S. pausiflorum* leaf indicated that there is no positive or negative significant (P < 0.05) correlation observed between any inorganic ions based on their preliminary data collected during the three different seasons. The K^+^ was positive and non-significantly (P > 0.05) correlated with Ca^2+^ (r = 0.579), Mg^2+^ (r = 0.225), Na^+^ (r = 0.065) and Cl^-^ (r = 0.658). The negative and non-significant correlation observed between the Ca^2+^ with Cl^–^ (r = − 0.078), Na^+^ (r = − 0.499) and Mg^2+^ (r = − 0.012); also, the Mg^2+^ negative and non-significantly correlated with Na^+^ (r = − 0.325). The Pearson correlation of the *S. persica* plant ionic constitute and indicated a negative and significant correlation (P < 0.05) in Na^+^ with K^+^ (r = − 0.716). The Na^+^ was negative and non-significantly (P > 0.05) correlated with Ca^2+^ (r = 0.615); Except Na^+^ with K^+^ and Ca^2+^ all other ionic constitutes were positive and non-significant.

### Major findings

The correlations between different ions in the leaves varied across plant species, indicating species-specific differences in ion regulation and interaction. While some elements showed significant correlations, others did not, suggesting complex regulatory mechanisms governing ion composition in plant leaves**.**

#### Internal ion regulation

Across all plant species studied, there is evidence of internal ion regulation, with certain ions showing consistent positive correlations, such as Ca^2+^ with Na^+^, K^+^, and Mg^2+^ in *H. mucronatum*, and Na^+^ with K^+^ in *C. conglomeratus.*

#### Species-specific differences

Each plant species demonstrates unique patterns of ion interactions. For instance, while Cl^-^ does not show significant correlations with other ions in *H. mucronatum* and *C. conglomeratus*, it exhibits a positive but non-significant correlation with K^+^ in *S. pausiflorum.*

#### Complex regulatory mechanisms

The presence of both significant and non-significant correlations suggests complex regulatory mechanisms governing ion composition in plant leaves. These mechanisms likely involve intricate interactions between various ions and physiological processes within each plant species.

#### Role of environmental factors

The absence of significant correlations in some cases, such as in *S. pausiflorum,* may indicate the influence of environmental factors or seasonal variations on ion regulation. Further investigation into the impact of environmental conditions on ion composition could provide additional insights.

### ANOVA and interclass correlation (ICC)

The results of an analysis of variance (ANOVA) and interclass correlation (ICC) analysis conducted on Ash, pH, and EC (electrical conductivity) parameters in the selected location of *Halopyrum mucronate* (Table 6). The ANOVA results indicate significant variations among raters for Ash (F = 152.6, p < 0.05), whereas no significant variations were observed between cases (F = 0.9471, p > 0.05). The within-case analysis revealed a substantial variation for all parameters, with mean squares ranging from 1.69245 to 40.4949. The provided models, labeled as Model 1, Model 2, and Model 3, represent different analyses or variations of the interclass correlation (ICC) calculations for EC, pH, and ash content of *Halopyrum mucronatum*. Each model focuses on different aspects of the ICC calculations. ICC is a widely used statistical measure to evaluate the reliability and consistency of measurements. By applying ICC to the measurements of EC, pH, and ash content, this study can determine the extent to which the measurements are consistent within the same location (individual ICC) and across different locations (mean ICC). It provides insights into the reliability of the measurements and the degree of agreement among raters or cases.

**Table 6:** ANOVA and interclass correlation (ICC) analysis of *Halopyrum mucronatum.*

Sum of sqrs	df	Mean square	F	
Between raters	545.485	2	272.742	152.6
Between cases	10.1547	6	1.69245	0.9471
Within cases	566.929	14	40.4949	
Residual	21.4438	12	1.78698	
Total	577.083	20		

				95% confidence
Model 1	Individual	ICC (1, 1)	− 0.4693	[− 0.4911, − 0.3505]
	Mean	ICC (1, k)	− 22.93	[− 82.78, − 3.517]
Model 2	Individual	ICC (2, 1)	− 0.0007787	[− 0.0113, 0.0574]
	Mean	ICC (2, k)	− 0.00234	[− 0.0347, 0.1545]
Model 3	Individual	ICC (3, 1)	− 0.01795	[− 0.3309, 0.5764]
	Mean	ICC (3, k)	− 0.05585	[− 2.937, 0.8032]

In Model 1, individual ICC (ICC (1, 1)): This value represents the ICC calculated for each specific variable (EC, pH, and ash content) within the same location. The values reported are − 0.4693 for EC, − 0.4693 for pH, and − 0.4693 for ash content. Mean ICC (ICC (1, k)): This value represents the average ICC calculated across all locations for each variable. The reported mean ICC values are − 22.93 for EC, − 22.93 for pH, and − 22.93 for ash content. In Model 2, individual ICC (ICC (2, 1)) value represents the ICC calculated for each specific variable within the same location. The reported values are − 0.0007787 for EC, − 0.0007787 for pH, and − 0.0007787 for ash content. Mean ICC (ICC (2, k)): This value represents the average ICC calculated across all locations for each variable. The reported mean ICC values are − 0.00234 for EC, − 0.00234 for pH, and − 0.00234 for ash content. In Model 3, individual ICC (ICC (3, 1)): This value represents the ICC calculated for each specific variable within the same location. The reported values are − 0.01795 for EC, − 0.01795 for pH, and − 0.01795 for ash content. Mean ICC (ICC (3, k)): This value represents the average ICC calculated across all locations for each variable. The reported mean ICC values are − 0.05585 for EC, − 0.05585 for pH, and − 0.05585 for ash content.

In *Cyprus conglomeratus*, the results of an analysis of the EC (electrical conductivity), pH (potential of hydrogen), and ash content were reported from various locations (Table 7). The statistical analysis includes calculations such as the sum of squares, degrees of freedom, mean square, and F-value, which are used to assess the variability and significance of the measured parameters. In terms of the variability between raters, the analysis indicates a sum of squares of 905.949 with 2 degrees of freedom, resulting in a mean square of 452.974. The calculated F-value of 218 suggests a significant difference between the raters in terms of their evaluations of the collected samples. Regarding the variability between cases, the sum of squares is reported as 22.621, with 10 degrees of freedom, leading to a mean square of 2.2621. The associated F-value of 1.089 indicates a relatively small difference between the cases in terms of the measured parameters. The within-case variability is represented by a sum of squares of 947.498 with 22 degrees of freedom, resulting in a mean square of 43.0681. This within-case variability reflects the natural variation of the measured parameters within each individual sample. The residual variability, which represents the unexplained variability after accounting for the other sources of variation, is reported as 41.5494 with 20 degrees of freedom, resulting in a mean square of 2.07747. This residual variability could be due to measurement error or other unaccounted factors. The total variability, obtained by summing the between-case, within-case, and residual variabilities, is reported as 970.119 with 32 degrees of freedom.

**Table 7:** ANOVA and interclass correlation (ICC) analysis of *Cyprus conglomeratus*.

Sum of sqrs	df	Mean square	F	
Between raters	905.949	2	452.974	218
Between cases	22.621	10	2.2621	1.089
Within cases	947.498	22	43.0681	
Residual	41.5494	20	2.07747	
Total	970.119	32		

				95% confidence
Model 1	Individual	ICC (1, 1)	− 0.4616	[− 0.4855, − 0.3774]
	Mean	ICC (1, k)	− 18.04	[− 50.4, − 4.617]
Model 2	Individual	ICC (2, 1)	0.001427	[− 0.009691, 0.04148]
	Mean	ICC (2, k)	0.004269	[− 0.02965, 0.1149]
Model 3	Individual	ICC (3, 1)	0.02877	[− 0.2539, 0.4757]
	Mean	ICC (3, k)	0.08162	[− 1.547, 0.7314]

To assess the agreement or consistency between the raters and the overall mean, the intraclass correlation coefficients (ICCs) are calculated. In Model 1, the ICC (1, 1) for individual raters is − 0.4616, indicating poor agreement among the raters for evaluating the collected samples. Similarly, the ICC (1, k) for the mean of all raters is − 18.04, suggesting substantial inconsistency in the overall mean measurements. In Model 2, the ICC (2, 1) for individual raters is 0.001427, indicating minimal agreement among the raters. The ICC (2, k) for the mean of all raters is 0.004269, further supporting the lack of agreement in the overall mean measurements. In Model 3, the ICC(3, 1) for individual raters is 0.02877, suggesting a slightly higher level of agreement among the raters compared to the previous models. The ICC (3, k) for the mean of all raters is 0.08162, indicating a slight improvement in the agreement for the overall mean measurements.

The results of an analysis of the EC (electrical conductivity), pH (potential of hydrogen), and ash content of *Sericostoma pauciflorum* samples collected from different locations (Table 8). Similar to the previous example, the statistical analysis includes calculations such as the sum of squares, degrees of freedom, mean square, and F-value to assess the variability and significance of the measured parameters. In terms of the variability between raters, the analysis indicates a sum of squares of 1931.01 with 2 degrees of freedom, resulting in a mean square of 965.504. The calculated F-value of 38.61 suggests a significant difference between the raters in terms of their evaluations of the collected samples. Regarding the variability between cases, the sum of squares is reported as 124.191, with 5 degrees of freedom, leading to a mean square of 24.8381. The associated F-value of 0.9934 indicates a relatively small difference between the cases in terms of the measured parameters. The within-case variability is represented by a sum of squares of 2181.05 with 12 degrees of freedom, resulting in a mean square of 181.754. This within-case variability reflects the natural variation of the measured parameters within each individual sample. The residual variability, which represents the unexplained variability after accounting for the other sources of variation, is reported as 250.044 with 10 degrees of freedom, resulting in a mean square of 25.0044. This residual variability could be due to measurement error or other unaccounted factors. The total variability, obtained by summing the between-case, within-case, and residual variabilities, is reported as 2305.24 with 17 degrees of freedom.

**Table 8:** ANOVA and interclass correlation (ICC) analysis of *Sericostoma pauciflorum*.

Sum of sqrs	df	Mean square	F	
Between raters	1931.01	2	965.504	38.61
Between cases	124.191	5	24.8381	0.9934
Within cases	2181.05	12	181.754	
Residual	250.044	10	25.0044	
Total	2305.24	17		

				95% confidence
Model 1	Individual	ICC (1, 1)	− 0.4041	[− 0.4741, − 0.03748]
	Mean	ICC (1, k)	− 6.318	[− 27.47, − 0.1215]
Model 2	Individual	ICC (2, 1)	− 0.000305	[− 0.03645, 0.2038]
	Mean	ICC (2, k)	− 0.0009156	[− 0.118, 0.4344]
Model 3	Individual	ICC (3, 1)	− 0.002221	[− 0.3426, 0.6502]
	Mean	ICC (3, k)	− 0.006694	[− 3.264, 0.8479]

To assess the agreement or consistency between the raters and the overall mean, the Intraclass Correlation Coefficients (ICCs) are calculated. In Model 1, the ICC (1, 1) for individual raters is − 0.4041, indicating poor agreement among the raters for evaluating the collected samples. Similarly, the ICC (1, k) for the mean of all raters is -6.318, suggesting substantial inconsistency in the overall mean measurements. In Model 2, the ICC (2, 1) for individual raters is -0.000305, indicating minimal agreement among the raters. The ICC (2, k) for the mean of all raters is -0.0009156, further supporting the lack of agreement in the overall mean measurements. In Model 3, the ICC (3, 1) for individual raters is − 0.002221, suggesting a slightly higher level of agreement among the raters compared to the previous models. The ICC (3, k) for the mean of all raters is -0.006694, indicating a slight improvement in the agreement for the overall mean measurements.

The results (Table 9) provide important information about the pH, EC (electrical conductivity), and ash content of *Salvadora persica* samples collected from different locations. It helps us understand how these properties vary among the samples and how reliable the measurements. Regarding the variability between raters, a significant difference was observed (F = 36.3, p < 0.05), as indicated by the sum of squares of 787.985 and 2 degrees of freedom. This suggests that the raters' evaluations of the collected samples exhibited considerable variation for the measured parameters. In terms of the variability between cases, there was a relatively small difference (F = 1.045, p > 0.05), as reflected by the sum of squares of 34.0223 and 3 degrees of freedom. This indicates that the samples collected from different locations had comparable measurements for the investigated parameters. The within-case variability, which represents the inherent variation within each individual sample, was assessed through the sum of squares of 853.112 with 8 degrees of freedom. The total variability, obtained by summing the between-rater, between-case, and within-case variabilities, was 887.134 with 11 degrees of freedom.

**Table 9:** ANOVA and interclass correlation (ICC) analysis of *Salvadora persica.*

Sum of sqrs	df	Mean square	F	
Between raters	787.985	2	393.993	36.3
Between cases	34.0223	3	11.3408	1.045
Within cases	853.112	8	106.639	
Residual	65.1265	6	10.8544	
Total	887.134	11		

				95% confidence
Model 1	Individual	ICC (1, 1)	− 0.4243	[− 0.4854, 0.154]
	Mean	ICC (1, k)	− 8.403	[− 49.93, 0.3533]
Model 2	Individual	ICC (2, 1)	0.001518	[− 0.02905, 0.3272]
	Mean	ICC (2, k)	0.00454	[− 0.09254, 0.5934]
Model 3	Individual	ICC (3, 1)	0.01472	[− 0.39, 0.8275]
	Mean	ICC (3, k)	0.04289	[− 5.316, 0.935]

Model 1 examines the agreement or consistency among the raters in evaluating the pH, EC, and ash content of the *Salvadora persica* samples. The Individual ICC (1, 1) value of − 0.4243 indicates poor agreement among the raters, implying that their evaluations of the samples show significant variability. The 95% confidence interval [− 0.4854, 0.154] further confirms the uncertainty in their agreement. Similarly, the Mean ICC(1, k) value of − 8.403, along with the 95% confidence interval [− 49.93, 0.3533], indicates a substantial inconsistency in the overall mean measurements. This suggests that the raters' assessments of the samples differ significantly, leading to inconsistency in the average values derived from their evaluations. These results emphasize the need for improved agreement and reliability among raters when assessing the pH, EC, and ash content of *Salvadora persica* samples.

In Model 2, the focus is on assessing the agreement or consistency among raters and the overall mean measurements for the pH, EC, and ash content of the *Salvadora persica* samples. The Individual ICC (2, 1) value of 0.001518 suggests minimal agreement among the raters, indicating that their evaluations show only slight consistency. The 95% confidence interval [− 0.02905, 0.3272] further underscores the uncertainty in their agreement. Similarly, the Mean ICC (2, k) value of 0.00454, along with the 95% confidence interval [− 0.09254, 0.5934], indicates limited agreement in the overall mean measurements. This implies that the raters' assessments of the samples and the average values derived from their evaluations exhibit only slight consistency. Therefore, there is room for improvement in achieving a higher level of agreement among the raters and in obtaining more consistent mean measurements for these parameters.

Model 3 aims to determine the agreement or consistency among raters and the overall mean measurements for the pH, EC, and ash content of the *Salvadora persica* samples. The Individual ICC (3, 1) value of 0.01472 suggests a slightly higher level of agreement among the raters compared to the previous models. However, the 95% confidence interval [− 0.39, 0.8275] indicates some uncertainty in their agreement. The Mean ICC (3, k) value of 0.04289, along with the 95% confidence interval [− 5.316, 0.935], suggests a slight improvement in the agreement for the overall mean measurements. While there is some consistency observed among the raters and in the mean measurements, there are still variations and uncertainties present. These findings emphasize the importance of further enhancing the agreement among raters and striving for more consistent mean measurements for accurate assessment of the pH, EC, and ash content of *Salvadora persica* samples.

### Major findings

The significance of the study lies in its contribution to several key areas within plant science and research methodology:

#### Understanding plant physiology

By analyzing the variability and correlations in the ionic composition and other parameters of different plant species, the study provides insights into the underlying physiological processes governing plant growth, development, and adaptation to environmental conditions. This understanding is crucial for advancing agricultural practices, conservation efforts, and ecosystem management.

#### Quality assurance in data collection

The study highlights the importance of standardized measurement protocols and rigorous quality control measures in ensuring the reliability and consistency of data collected in plant science research. By identifying sources of variability and potential measurement errors, the study underscores the need for systematic approaches to data collection and analysis.

#### Enhancing RESEARCH methodology

Through the application of statistical analyses such as ANOVA and ICC, the study demonstrates the use of advanced research methodologies to assess variability, correlations, and agreement in plant parameters measured by multiple raters or across different locations. These methodologies can serve as valuable tools for researchers in designing experiments, analyzing data, and drawing meaningful conclusions from their findings.

#### Informing agricultural practices

The findings of the study have practical implications for agricultural management, as they provide insights into the factors influencing the nutrient content, pH levels, and other important parameters in plant species. This information can guide farmers and agricultural practitioners in making informed decisions related to crop selection, soil fertility management, and nutrient supplementation.

#### Supporting environmental conservation

By studying the variability and correlations in plant parameters across different locations, the study contributes to our understanding of ecosystem dynamics and biodiversity conservation. This knowledge can inform conservation strategies aimed at protecting plant species and their habitats in the face of environmental challenges such as climate change and habitat loss.

#### Facilitating future prospectives

The study lays the groundwork for future research endeavors by identifying areas for further investigation and refinement. By highlighting the limitations and challenges associated with current methodologies, the study paves the way for future studies aimed at addressing these issues and advancing our understanding of plant biology and ecology.

In summary, the significance of the study lies in its contribution to advancing knowledge in plant science, improving research methodology, informing agricultural practices, supporting environmental conservation efforts, and laying the groundwork for future research endeavors in these important areas.

## Discussion

The study presents a comprehensive analysis of heavy metal bioavailability and mineral characterization in halophytic plants using multivariate statistical techniques. Here, we discuss the findings of the study along with relevant literature to contextualize the results and implications. The study likely employed multivariate statistical methods such as principal component analysis (PCA) to identify patterns of heavy metal bioavailability in halophytic plants. PCA might have been used to reduce the dimensionality of the dataset, identifying dominant factors influencing heavy metal uptake and accumulation in different plant species. Calone et al.^38^ performed the PCA in six halophytic species in control and control normalized PCA to 74.4% and 72.2% of total variance, respectively; however, 99.65% of total variance were observed through PCA in selected flora in this study. Wuana and Okieimen^39^ and Zeng et al.^40^ discuss the complex mechanisms governing heavy metal uptake in plants, emphasizing the importance of understanding soil–plant interactions and physiological processes. Song et. al.^41^ was statistically analyzed the element concentration in the leaf of halophytic species with the soil elements concentration and recorded a significant (P < 0.001) positive correlation between Ca^2+^ with Mg^2+^ and K^+^ in the soil elements; however, leaf K^+^ was positive and significantly (P < 0.001) correlated with leaf Ca^2+^ while the leaf Na^+^ was negative and significantly (P < 0.001) correlated with K^+^ and Ca^2+^. It was also recorded that there was a non-significantly (P > 0.05) correlated soil mineral with leaf ionic components but soil minerals were also significantly (P < 0.05; P < 0.01 and P < 0.001) correlated with leaf heavy metals. Also, it was analyzed ANOVA in mineral concentration with salt-tolerance type and plant parts (leaf, stem and root) and reported significant effects on the concentration of Na^+^(F = 14.88; P < 0.001), K^+^ (F = 4.90; P < 0.05), Ca^2+^ (F = 9.68; P < 0.01) and Mg^2+^ (F = 10.14; P < 0.01) in the salt-tolerance type; however, the plant parts were significantly affected by Na^+^(F = 14.07; P < 0.001), Ca^2+^ (F = 3.09; P < 0.05) and Mg^2+^ (F = 21.71; P < 0.001) concentration and non-significant affected by K^+^ (F = 0.82; P > 0.05) content. In the dicot plants, a very highly significant difference was observed in ash content as well as Na^+^ and Cl^-^ content and significant observed in Ca^2+^ at the species level and non-significant relation was observed at the seasonal level; however, in the monocot species, the significant difference observed only Ca^2+^ in the seasonal level^42^. The research paper provides valuable insights into the bioavailability of heavy metals and mineral characterization in halophytic plants using multivariate statistical analysis. By elucidating the mechanisms governing metal uptake and plant adaptation in saline environments, the study contributes to our understanding of halophyte ecology and their potential applications in phytoremediation and environmental management.

## Conclusion

In conclusion, this research paper examined the variability and agreement among raters in evaluating different plant species, namely *Halopyrum mucronatum, Cyprus conglomeratus, Sericostoma pauciflorum, and Salvadora persica.* The findings from the principal component analysis (PCA) highlighted the significant contributions of certain heavy metals (Cu, Fe, Mn) to the observed variance in each species, emphasizing their influential roles in determining the variability in heavy metal concentrations. The selected plant shows a significant correlation between some mineral constituents like *Halopyrum mucronatum* all macronutrients show a positive significant correlation except Cl^–^; while *Sericostoma pauciflorum* does not show any significance of the two variables. Furthermore, the ANOVA and intraclass correlation coefficients (ICC) analyses revealed valuable insights into the consistency and reliability of measurements within and between raters. While there may be room for improvement in achieving higher levels of agreement among raters, the results indicate the potential for enhancing measurement consistency in future evaluations. The differences between cases were relatively small, indicating that the measured parameters showed comparable values across different locations. These findings highlight the importance of ongoing efforts to address inter-rater variability and promote greater agreement among raters, ultimately contributing to more reliable assessments of the samples. Overall, this study underscores the importance of addressing inter-rater variability and enhancing measurement consistency when evaluating plant species. It highlights the influential heavy metals and their correlations, providing valuable information for future research and management of these plant species. These findings contribute to the understanding of the variability and reliability of measurements in the context of ecological and environmental studies, and emphasize the need for standardized protocols and improved agreement among raters to ensure accurate and consistent assessments.

## Data Availability

The datasets used and/or analysed during the current study available from the corresponding author on reasonable request.
